# Mechanical Behavior of Closed-Cell Ethylene-Vinyl Acetate Foam under Compression

**DOI:** 10.3390/polym16010034

**Published:** 2023-12-21

**Authors:** Hongjuan Chen, Deqiang Sun, Lulu Gao, Xiaochen Liu, Meilin Zhang

**Affiliations:** 1 College of Art and Design, Shaanxi University of Science & Technology, Xi’an 710021, China; chenhongjuan@sust.edu.cn; 2 College of Bioresources Chemical and Materials Engineering, Shaanxi University of Science & Technology, Xi’an 710021, China; 210112137@sust.edu.cn (L.G.); liuxiaochen@sust.edu.cn (X.L.); 202101030111@sust.edu.cn (M.Z.)

**Keywords:** closed-cell ethylene-vinyl acetate foam, static and dynamic compressions, density, strain rate, stress−strain curve, energy absorption efficiency, energy absorption diagram

## Abstract

The static and dynamic compressions of closed-cell ethylene-vinyl acetate (EVA) foams with different densities were conducted under various strain rates. The stress−strain curves were processed to determine the corresponding curves of energy absorption per unit volume and energy absorption efficiency, and energy absorption diagrams were produced. The influences of density and strain rate on the elastic modulus, yield strength, energy absorption per unit volume, optimal strain, densification strain, and energy absorption diagrams were analyzed and discussed. The whole stress−strain curve can be fitted with the Rusch formula. The strain rate does not change the shape of stress−strain curve, and has little influence on the elastic modulus. There exists the optimal density of EVA foam corresponding to its maximum energy absorption efficiency. Under a fixed strain rate, the optical energy absorption per unit volume is proportional to the optical stress on the envelope line in the energy absorption diagrams of EVA foams with different densities. The change in strain rate leads to the envelope line in the energy absorption diagrams of EVA foams with a given density having the larger slope and a negative intercept where the optical energy absorption per unit volume relies linearly on the optical stress. The empirical formulas of elastic modulus, yield strength, optimal strain, and envelope lines and their slopes are derived from the tested results.

## 1. Introduction

EVA foam has excellent characteristics of energy absorption, cushioning, vibration isolation, resilience, being moisture proof, and heat insulation [1]. Therefore, it can be widely used as a protective material and cushioning packaging for various electronic devices, photovoltaic panels, valuables, high-precision instruments, etc. It is of great significance to study the mechanical behaviors of EVA foam for promoting its rational use.

The density determines the mechanical properties of foam materials. Recently, Rumianek et al. [2] studied the influence of material density on the static compressive strength and energy absorption capacity for closed-cell expanded polypropylene foams. Lutfi et al. [3] reported the compression and tension behaviors of polyurethane, EVA, Pelite™, and a combination of polyurethane and EVA with a given medium density. Xin [4] and Wen et al. [5] explored the mechanical performance and life prediction of EVA foams with five densities of 132, 151, 172, 188, and 209 kg/m^3^ under repetitive compressions. Duncan et al. [6] highlighted that the mechanical properties of foam materials are closely related to the compressive strain rate. Therefore, some scholars carried out some investigations of EVA foam under dynamic loadings. Liu et al. [7] reported the cushioning performance of EVA foam with a given density of 210 kg/m^3^ under different impact velocities. Ye et al. [8] analyzed the energy absorption performance of expanded polyethylene and EVA foams with three densities under dynamic impact. Additionally, Verdejo and Mills [9] pointed out that the air compression in EVA foams with the density range of 150–250 kg/m^3^ provides its main shock-absorption mechanism. Furthermore, Lam et al. [10] revealed that that thickness affected the cushioning performance of EVA foam with a given density of 120 kg/m^3^ under six successive impacts. In fact, under a certain loading velocity, changes in foam thickness produce different strain rates, which in turn, affect the foam’s mechanical behavior. It has been demonstrated that the loading condition is quasi-static compression when evaluating the cushioning performance of foams based on the test standards [11,12,13,14], while the loadings in actual cushioning applications are always dynamic. Therefore, the compressive strain rate must be considered in order to reveal the cushioning performance of EVA foams. Surely, temperature affects the mechanical properties of polymeric foams, and their thermo-physical properties have the inherent variation uncertainty with increasing temperatures [15]. However, the temperature change is always limited in packaging applications. So, temperature was not considered in this investigation.

By taking the influence of strain rate into account, and based on the energy absorption diagram method used by Zhang et al. [16], in this study, the cushioning performance evaluation method and the evaluation indicators were established to evaluate the energy absorption capability of closed-cell EVA foams. By discussing the influences of density and strain rate on the stress−strain curves, energy absorption efficiency, and energy absorption diagrams under compressions, the comprehensive effects of these factors on the static and dynamic mechanical properties of EVA foams are discovered so as to promote their reasonable utilization in cushioning packaging design.

## 2. Experimental Principles

### 2.1. Test Standards

All experiments were carried out according to the following test standards: (1) ASTM D1621-16, standard test method for the compressive properties of rigid cellular plastics [11]; (2) ISO 844, cellular plastics—compression test of rigid materials [12]; (3) GB/T 8813-2020, rigid cellular plastics—determination of compression properties [13]; and (4) GBT 8168-2008, testing method of static compression for packaging cushioning materials [14].

### 2.2. Specimens

Raw materials of EVA foam include the main ingredient, foaming agents, crosslinking agents, fillers, and functional additives. The main ingredient is an EVA copolymer, usually containing some quantity of polyethylene, and a small amount of polyene elastomer, ethylene propylene diene monomer, etc. There are four types of EVA foaming processes: molding foaming, injection foaming, continuous foaming, and extrusion foaming. All EVA foams used in this study were manufactured by the molding foaming method, and its basic manufacturing process includes eight steps of pretreatment, internal mixing, refining, sheet production, vulcanization, cooling, slicing, and packaging. Pretreatment involves the break-up, cleaning, and drying of raw materials. Internal mixing is the process of mixing and dispersing raw materials uniformly over a certain period of time at a certain temperature and pressure level. Refining involves further mixing to ensure the even dispersion of raw materials. During the sheet production process, the refined raw materials are made into sheets and cooled, and then cut according to the mold specification. Vulcanization is the process of crosslinking and foaming material sheets into the required specification within the mold at a certain temperature and pressure level for a certain period of time, which determines the final EVA foam density. Slicing is the process of cooling and shaping, and then slicing the RVA foam according to the requested thickness specifications.

**Figure 1:**
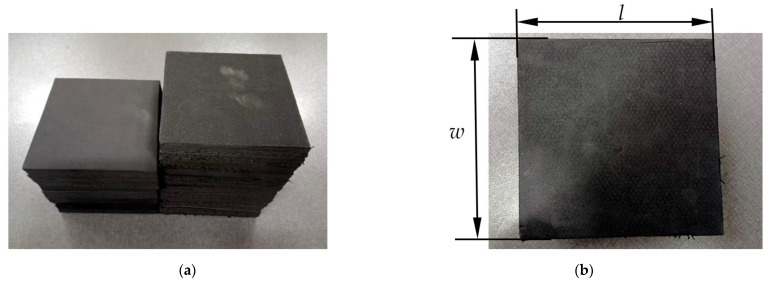
EVA foam specimens and dimensions: (**a**) Specimens with different densities; (**b**) Single specimen and its dimensions.

All EVA foam materials were purchased from the Dongguan Jingzhan Novel Material Co., Ltd. (Dongguan, China) with the consistent material prescription. The EVA foam materials with five different densities of 80, 95, 106, 124, and 180 kg/m^3^ commonly used in commerce were supplied from the same production batch. The EVA materials were cut by a special foam plate cutter to produce the specimens shown in Figure 1. Assuming that the length, width, and height of the single EVA foam specimens were *l*, *w*, and *h*, respectively, hereby, *l* = 100 mm, *w* = 100 mm, and *h* ≥ 30 mm, consistent with the above test standards.

### 2.3. Testing Devices

**Figure 2:**
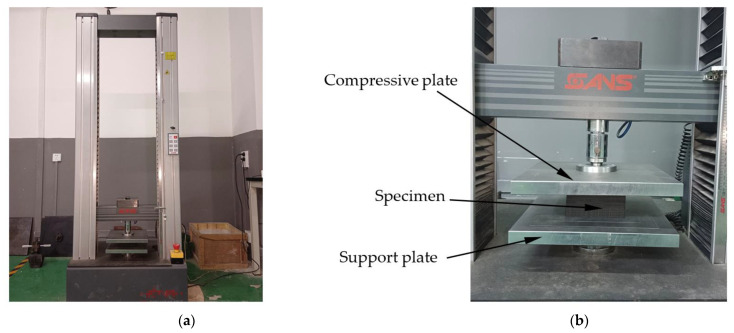
Testing machine used for compression of EVA foam specimen: (**a**) Universal material testing machine; (**b**) Specimen in compression.

All experimental machines used here and their applications were as follows: (1) As is shown in Figure 2a, the CMT4303 universal material testing machine with the loading capacity of 30 kN produced by the MTS System Corporation (Shanghai, China) was used for specimen compression (Figure 2b); (2) The AOL-1625-S foam plate cutter produced by Jinan Aolei CNC Equipment Co., Ltd. (Jinan, China) was used to make the EVA specimens; (3) The HWS-350 constant-temperature and humidity chamber produced by Beijing Zhongxing Weiye Century Instrument Co., Ltd. (Beijing, China) was used for the treatment of specimens; (4) The SL01-3 carbon fiber vernier caliper produced by Deqing Shengxin Electronic Technology Co., Ltd. (Huzhou, China) was used for specimen dimension testing.

### 2.4. Experimental Schemes

In accordance with the test standard GB/T 4857.2-2005 [17], all EVA foam specimens were pretreated in the HWS-350 constant-temperature and humidity chamber at a temperature of 23 °C and a relative humidity of 50% for more than 24 h. Subsequently, the compressions of EVA foam specimens were conducted by the CMT4303 universal material testing machine under the same temperature and humidity conditions.

The EVA foams with the above five densities were all employed. The velocity of the compressive plate of the universal material testing machine is assumed as *v*; then, the compressive strain rate $\dot{\varepsilon }$ is:

(1) $$\dot{\varepsilon }=v/h$$

For studying the quasi-static mechanical performance of EVA foams, all compression tests were carried out under the compressive strain rate of 0.02 min^−1^. When studying the influence of the compressive strain rate, the EVA foam specimens with a fixed density were compressed under eight different compressive strain rates from 0.02 min^−1^ to 25 min^−1^. To depict the complete energy absorption diagrams of EVA foams, the specimens with the above five densities were compressed under these eight different compressive strain rates. In all, at least forty specimens were employed.

## 3. Data analysis Methods

### 3.1. Generation of Response Curves

During the entire compression course, the support plate was fixed, and the upper and lower surfaces of the EVA specimens were pressed against the compressive and support plates (Figure 2b). It was assumed that the contact force of the compressive plate against the EVA specimen was *F*, and the reduction in specimen height was *u*. The *F* and *u* values were automatically recorded by the CMT4303 universal material testing machine. Then, the nominal stress *σ* and nominal strain *ε* were respectively defined as:

(2) $$\sigma =\frac{F}{l\times w},\;\varepsilon =\frac{u}{h}$$

**Figure 3:**
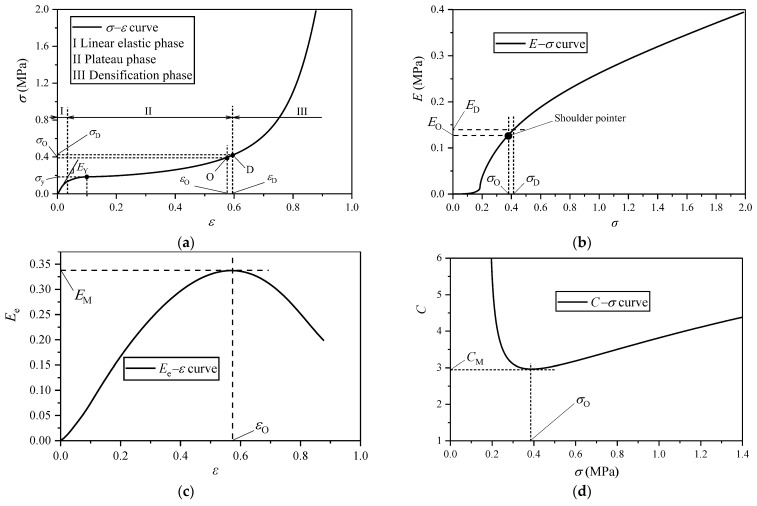
Typical response curves of EVA foam specimens under compression: (**a**) *σ*−*ε* curve; (**b**) *E*−*σ* curve; (**c**) *E*_e_−*ε* curve; (**d**) *C*−*σ* curve.

A typical compression *σ*−*ε* curve of EVA foam is shown in Figure 3a. By integrating the *σ*−*ε* curve, the energy absorption per unit volume *E* can be obtained as:

(3) $$E=\int_{0}^{\varepsilon }\sigma d\varepsilon$$

The corresponding *E*−*σ* curve is shown in Figure 3b. Miltz et al. [18] proposed to use the energy absorption efficiency *E*_e_ to characterize the energy absorption capacity of foam materials under a certain stress level of *σ*. *E*_e_ is defined as:

(4) $$E_{e}=\frac{\int_{0}^{\varepsilon }\sigma d\varepsilon }{\sigma }$$

The corresponding *E*_e_−*ε* curve is shown in Figure 3c. The reciprocal of *E*_e_ is called the cushioning coefficient *C,* as follows [19]:

(5) C=1Ee=σ∫0εσdε

The corresponding *C*−*σ* curve is shown in Figure 3d.

### 3.2. Equivalent Mechanical Model of Closed-Cell Foam

**Figure 4:**
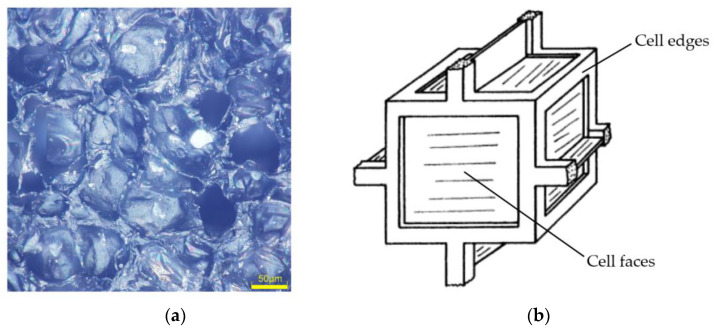
Mechanical model of EVA foam: (**a**) Microstructure observed in SEM; (**b**) Periodic cubic cell model [20].

The typical microstructure of closed-cell EVA foam observed with a scanning electron microscope (SEM) is shown in Figure 4a. Its mechanical model can be equivalent to the structure formed by infinitely expanding the periodic cubic cell model shown in Figure 4b in three-dimensional space [20]. The periodic cubic cell includes cell edges with square cross-section and six cell faces around it.

### 3.3. Evaluation Indicators

The typical *σ*−*ε* curve of EVA foam includes three deformation stages: linear elastic stage I, plateau stage II and densification stage III (Figure 3a). In the linear elastic stage, the stress relies on the strain approximately linearly with the slope called the elastic modulus *E*_Y_ [11,12,13] (Figure 3a). Then, the cells in the EVA foam undergo elastic buckling without a distinct yield point, followed by a non-linear plateau stage where the plateau stress appears (Figure 3a), since the contribution of fluid pressure inside the cells of EVA foam results in the stress strengthening with the increase in strain. For the EVA foams, there is no distinct yield point when *ε* ≤ 0.1, so the stress at *ε* = 0.1 is taken as the yield stress *σ*_y_ [11,12,13]. When the stress reaches a certain level, the energy absorption efficiency has a peak value (Figure 3c), which is called the maximum energy absorption efficiency *E*_M_; the corresponding cushioning coefficient has a minimum value (Figure 3d), which is named the minimum cushioning coefficient *C*_M_; this time, it means that the energy absorption capacity reaches the highest level, and the corresponding strain, stress, and energy absorptions per unit volume are called the optimal strain *ε*_O_, optimal stress *σ*_O_, and optimal energy absorption per unit volume *E*_O_, respectively (Figure 3a−c); the corresponding shoulder point appears on the *E*−*σ* curve (Figure 3b).

Similar to the general closed-cell foam materials, when the cells in EVA foam completely collapse with the cell faces and cell edges contacted together, EVA foam begins to enter the densification stage, and the corresponding strain and stress are called the densification strain *ε*_D_ and densification stress *σ*_D_, respectively (Figure 3a); afterwards, the stress increased sharply (Figure 3a). For a given EVA foam material, its optimal strain is smaller than its densification strain. After the compression load is removed, although the cell-wall base material of EVA foam exhibits plastic buckling, which results in permanent deformation, most deformation of the specimen will be recovered with the assistance of gas pressure in the foam. Therefore, the closed-cell EVA foam material can be regarded as an elastomer [20]. Under quasi-static compression, the following empirical relationship between *ε*_D_ and relative density *ρ*/*ρ*_s_ has been provided as follows [20]:

(6) $$\varepsilon_{D}=1-1.4\rho /\rho_{s}$$

where *ρ* is the density of EVA foam, and *ρ*_s_ is the density of the cell-wall base material of the EVA foam. The cell-wall base material is the EVA copolymer.

## 4. Results and Analysis

### 4.1. Stress−Strain Curves

#### 4.1.1. Influence of Density

**Figure 5:**
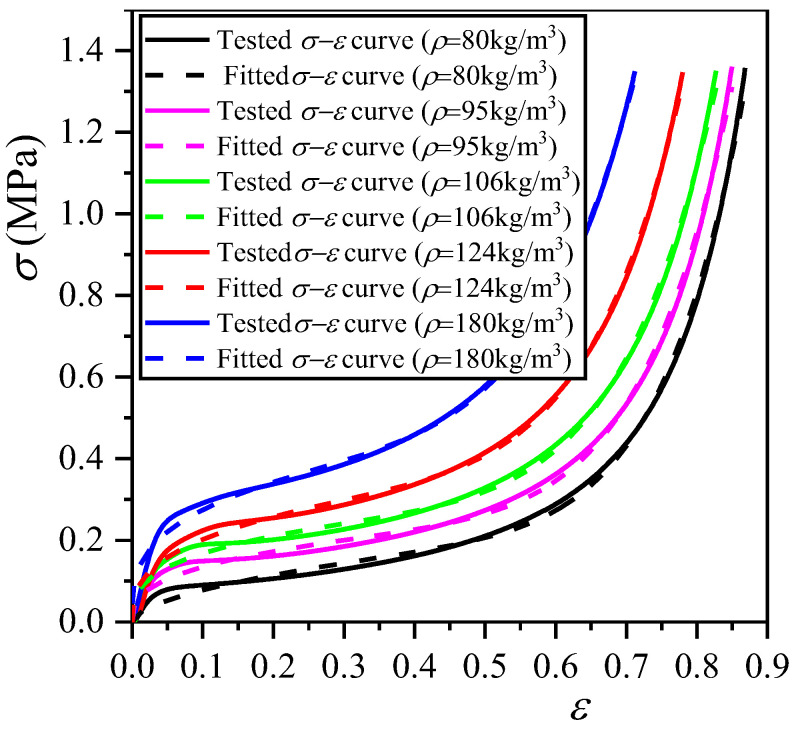
Typical *σ*−*ε* curves of EVA foam specimens with various densities under quasi-static compressions.

The quasi-static ($\dot{\varepsilon }$ = 0.02 min^−1^) compression tests were carried out for the closed-cell EVA foams (*h* = 50 mm) with the five different densities mentioned above, and the typical *σ*−*ε* curves are shown in Figure 5.

It can be seen from Figure 5 that the EVA foam with a higher density has a larger stress at the same strain, which means a higher elastic modulus, yield strength, and energy absorption. The constitutive equation reflects the stress−strain relationship of the material throughout the entire compression process. Under a certain strain rate, the constitutive equation of EVA foam with a given density can be fitted with the Rusch formula as follows [19]:

(7) $$\sigma =A\varepsilon^{m}+B\varepsilon^{n}$$

Using Equation (7) to fit the above curves, the fitted curves are plotted in Figure 5. For the EVA foams with densities of 80, 95, 106, 124, and 180 kg/m^3^, their constitutive equations are *σ* = 3.095*ε*^7.669^ + 0.281*ε*^0.557^, *σ* = 3.186*ε*^6.981^ + 0.307*ε*^0.357^, *σ* = 3.349*ε*^6.493^ + 0.354*ε*^0.323^, *σ* = 4.406*ε*^6.363^ + 0.452*ε*^0.349^, and *σ* = 5.386*ε*^5.532^ + 0.567*ε*^0.314^, respectively.

*E*_Y_ and *σ*_y_ are two important physical parameters indicating the static mechanics of polymer foams, and we hereby try to establish the relationship between them with their relevant factors. The corresponding *E*_Y_ and *σ*_y_ values can be calculated from the *σ*−*ε* curves in Figure 5, and these are listed in Table 1. The relationship between the *E*_Y_ of closed-cell EVA elastomer foam and *ρ*/*ρ*_s_ meets the following equation [20]:

(8) $$\frac{E_{Y}}{E_{s}}\approx \varphi^{2}{\left(\frac{\rho }{\rho_{s}}\right)}^{2}+\left(1-\varphi \right)\frac{\rho }{\rho_{s}}+\frac{p_{0}\left(1-2\nu \right)}{E_{s}\left(1-\rho /\rho_{s}\right)}$$

where *E*_s_ is the elastic modulus of the cell-wall base material in EVA foam and *ϕ* is the solid fraction of the cell edges of EVA foam; then, the solid fraction of the cell faces is 1−*ϕ*; *ν* is the relationship coefficient, *ν* ≈ 1/3; and *p*_0_ is the initial fluid pressure in the cells of EVA foam. This is generally close to or slightly larger than the atmospheric pressure *p*_at_, and relatively very small compared with *E*_s_, so the third item in the above Equation (8) can be ignored.

According to the mechanical parameters provided by the EVA supplier, *E*_s_ = 112 MPa and *ρ*_s_ = 950 kg/m^3^. For all EVA foams employed here, *ρ*/*ρ*_s_ < 0.2, so the relationship between *E*_Y_ and *ρ*/*ρ*_s_ also meets the following [21]:

(9) $$E_{Y}/E_{s}=0.32\left({\left(\rho /\rho_{s}\right)}^{2}+\rho /\rho_{s}\right)$$

For the EVA foams with a wider range of densities, assuming that the solid fraction of cell faces is 0, viz. *ϕ* = 1, Equation (8) is simplified as follows [20]:

(10) $$E_{Y}/E_{s}\approx {\left(\rho /\rho_{s}\right)}^{2}$$

**Figure 6:**
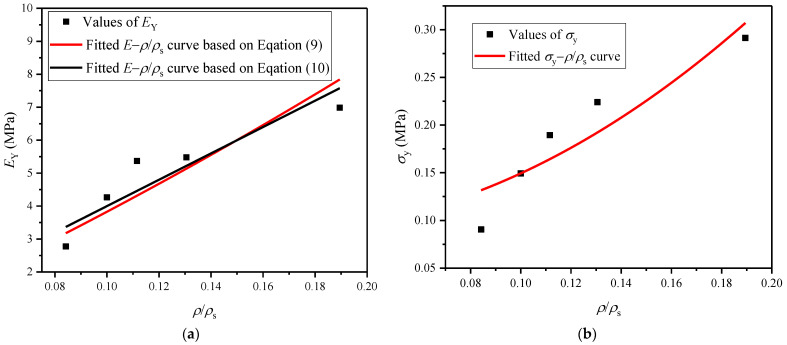
(**a**) *E*−*ρ*/*ρ*_s_ and (**b**) *σ*_y_−*ρ*/*ρ*_s_ curves of EVA foam specimens with different densities under quasi-static compression.

The tested *E*_Y_ values of EVA foams with five densities and the fitted *E*_Y_−*ρ*/*ρ*_s_ curves based on Equations (9) and (10) are shown in Figure 6a. Based on Equation (9), it can be fitted that *E*_s_ = 108.7252 MPa, which is consistent with the information provided by the EVA supplier, indicating that Equation (9) has the higher accuracy.

For the closed-cell EVA elastomer foam, the relationship between *σ*_y_ and *ρ*/*ρ*_s_ is as follows [20]:

(11) $$\sigma_{y}=0.05E_{s}{\left(\rho /\rho_{s}\right)}^{2}+\Delta p$$

where Δ*p* is the initial pressure difference between *p*_0_ and *p*_at_ in the cells of EVA foam before compression. The tested *σ*_y_ values of EVA foams with five densities and the fitted *σ*_y_−*ρ*/*ρ*_s_ curve are shown in Figure 6b. From the fitted *σ*_y_−*ρ*/*ρ*_s_ curve, it can be fitted that *E*_s_ = 121.7457 MPa, which is close to that given by EVA supplier. The fitted Δ*p* = 0.0885 MPa, indicating that *p*_0_ is surely slightly larger than *p*_at_.

#### 4.1.2. Influence of Strain Rate

**Figure 7:**
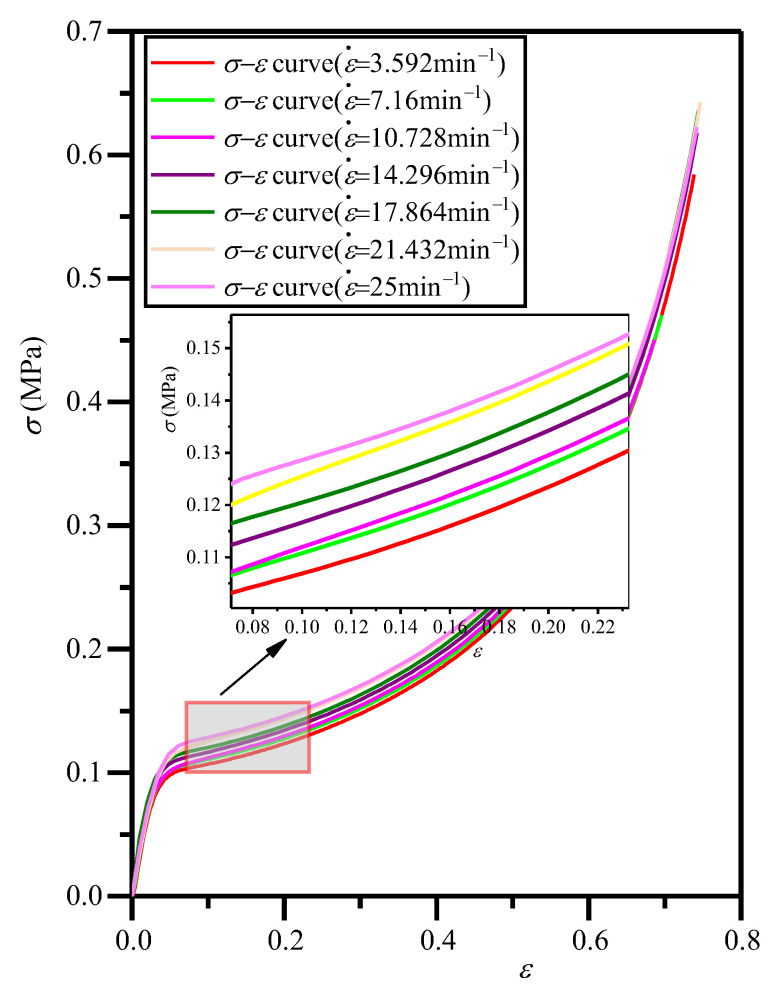
Typical *σ*−*ε* curves of EVA foam specimen specimens with a given density (*ρ* = 80 kg/m^3^) under various compressive strain rates.

In order to explore the effect of strain rate on the *σ*−*ε* curves, the EVA foam specimens with a fixed density (*ρ* = 80 kg/m^3^) were also compressed under the compressive strain rates of 3.592, 7.16, 10.728, 14.296, 17.864, 21.432, and 25 min^−1^, and the corresponding *σ*−*ε* curves are shown in Figure 7. The closed-cell EVA foams have a high strain rate sensitivity, mainly due to the strain rate sensitivity of the air in the cells of the EVA foam. From Figure 7, it can be seen that the strain rate has not obviously changed the shape of the *σ*−*ε* curve with three typical deformation stages. At the same strain, for the EVA foam specimens with a given density, the larger the strain rate, the higher the deformation velocity, and the greater the corresponding stress, yield stress, and energy absorption.

**Figure 8:**
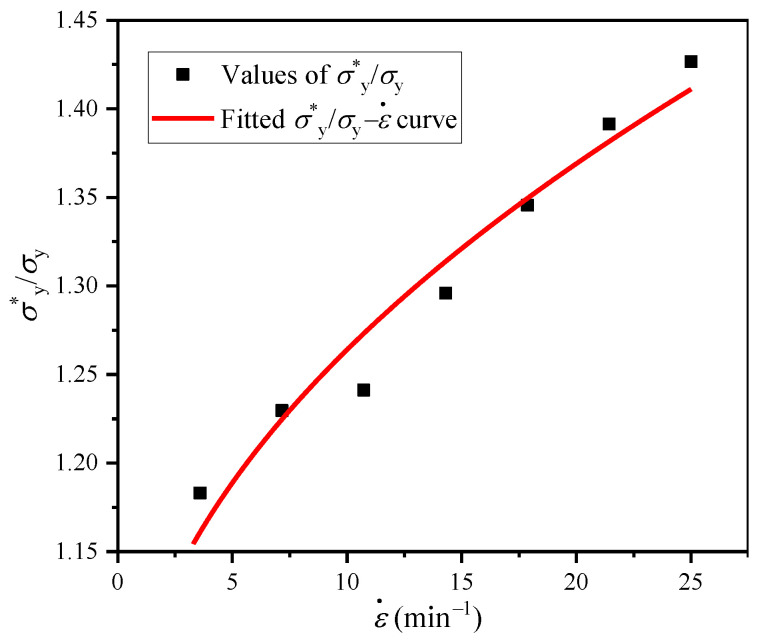
Typical σ*_y_/*σ*_y_−$\dot{\varepsilon }$ curves of EVA foam specimens with a given density (*ρ* = 80 kg/m^3^).

The values of dynamic elastic modulus E* and dynamic yield stress σ*_y_ under different strain rates can be obtained from the *σ*−*ε* curves in Figure 7, and they are listed in Table 2. It can be seen that the E* value of closed-cell EVA foam is not sensitive to strain rate. This is mainly attributable to the Young’s modulus of EVA foam material being mainly determined by the stretching and bending of cell edges and cell faces, with little dependence on the fluid pressure in the cells, as shown in Equation (8). Hereby, we introduce a strain rate improvement representing the increase in σ*_y_ compared to *σ*_y_ due to the increase in strain rate, which is the second term of the following equation [22]:

(12) $$\sigma^{*}{}_{y}/\sigma_{y}=1+B{\dot{\varepsilon }}^{P}$$

where the coefficient *B* and the exponent *P* are all material-related constants. For the EVA foam with *ρ* = 80 kg/m^3^, the quasi-static yield stress *σ*_y_ = 0.0905 MPa (seen in Table 1). Based on Equation (12), by fitting the σ*_y_/*σ*_y_−$\dot{\varepsilon }$ curve, it can be calculated that *B* = 0.0868 and *P* = 0.483, as shown in Figure 8. By combining Equations (11) and (12), the empirical formula of σ*_y_ for EVA foams can be obtained as:

(13) $$\sigma^{*}{}_{y}=\left(0.05E_{s}{\left(\rho /\rho_{s}\right)}^{2}+\Delta p\right)\left(1+0.0868{\dot{\varepsilon }}^{0.483}\right)$$

### 4.2. Energy Absorption Efficiency

#### 4.2.1. Influence of Density

**Figure 9:**
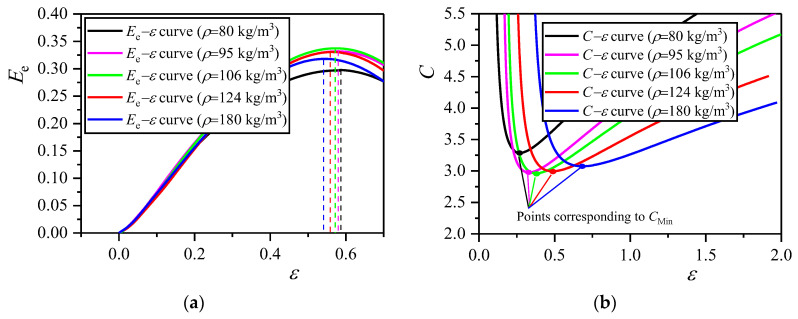
Energy absorption efficiency and cushioning coefficient of EVA foam specimens with different densities under quasi-static compression: (**a**) *E*_e_−*ε* curves; (**b**) *C*−*ε* curves.

The corresponding *E*_e_−*ε* and *C*−*ε* curves, obtained from the quasi-static *σ*−*ε* curves of closed-cell EVA foams with different densities (Figure 5), are plotted in Figure 9a,b. The corresponding *E*_M_ and *ε*_O_ values calculated from the *E*_e_−*ε* curves (Figure 9a) are listed in the second and third columns of Table 3. The corresponding *C*_M_ values calculated from the *C*−*ε* curves (Figure 9b) are listed in the fourth column of Table 3. As *ρ*_s_ = 950 kg/m^3^, according to Equation (6), the calculated *ε*_D_ value of EVA foams with different densities are listed in the last column of Table 3.

As mentioned above, *E*_M_ represents the maximum energy absorption capacity of EVA foams. The higher the *E*_M_ value, the stronger the energy absorption capacity; conversely, the smaller the *C*_M_ value, the stronger the energy absorption capacity, and the better the cushioning performance of EVA foams. Here we discover the dependence of maximum energy absorption efficiency on density under a certain strain rate. As is listed in Table 3, as the density of EVA foams increases, *E*_M_ first increases and then decreases. Meanwhile, *C*_M_ first decreases and then increases; when *ρ* approaches 106 kg/m^3^, *E*_M_ approaches the maximum value and *C*_M_ approaches the minimum value (Figure 9a,b). This means that the EVA foam has an optimal density corresponding to the largest *E*_M_ value.

**Figure 10:**
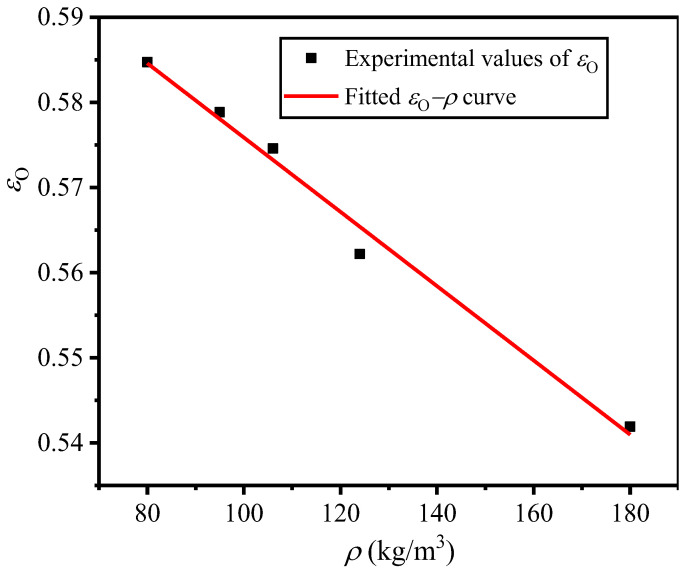
*ε*_O_−*ρ* curve of EVA foams under quasi-static compression.

It can also be seen that under a certain compressive strain rate, the *ε*_O_ value of EVA foam with a given density is smaller than its corresponding *ε*_D_ value. However, under a certain strain rate, the optical strain also depends on the density of EVA foam. Under a certain compressive strain rate, the EVA foam with a higher density has smaller *ε*_O_ and *ε*_D_ values, but *ε*_O_ is closer to *ε*_D_ with the increase in the density of the EVA foam. This is because the higher the density of EVA foam, the lower the internal porosity. In addition, densification occurs at the smaller strain, under a certain compressive strain rate. Similar to the densification strain in Equation (6), under quasi-static compression, the empirical formula of *ε*_O_ is expressed as:

(14) $$\varepsilon_{O}=\gamma_{O}-\lambda_{c}\rho /\rho_{s}$$

where *γ*_O_ is the ideal porosity of EVA foam with *ρ* = 0 kg/m^3^, with the theoretical value of 1; however, the actual tested value is far smaller than 1; *λ*_C_ is the relationship coefficient. Based on the quasi-static tested results in Table 3, using Equation (14), it can be fitted that *γ*_O_ = 0.6195 and *λ*_C_ = 0.4144, and the corresponding fitted curve is shown in Figure 10.

#### 4.2.2. Influence of Strain Rate

**Figure 11:**
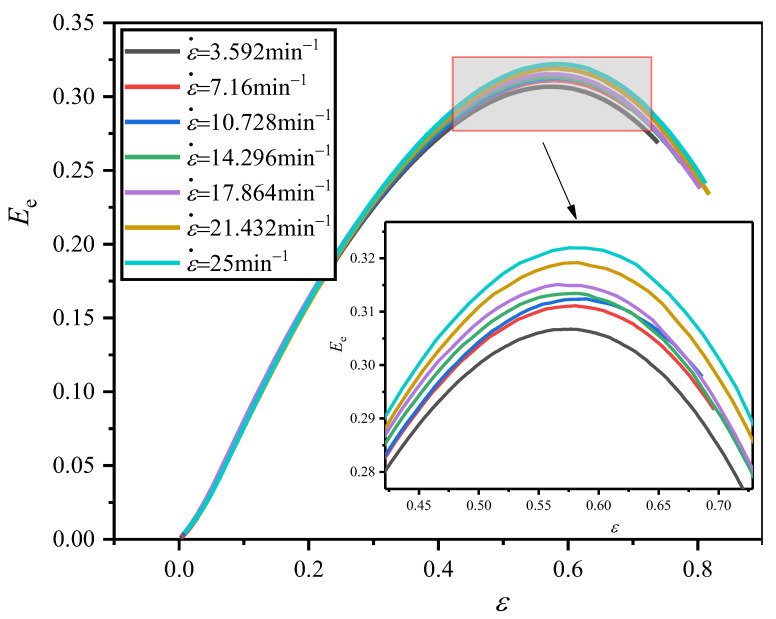
Typical *E*_e_−*ε* curves of EVA foam specimens with a given density (*ρ* = 80 kg/m^3^) under various compressive strain rates.

The corresponding *E*_e_−*ε* curves obtained from the *σ*−*ε* curves (Figure 7) of EVA foams, are shown in Figure 11. The corresponding *E*_M_ and *ε*_O_ values are obtained and listed in Table 4. From Figure 7, it is seen that the stress in the plateau stage at a certain strain increases with the increase in strain rate. Meanwhile, this is accompanied by the slight increase in optimal strain for the closed-cell EVA foam with a given density, which results in a higher energy absorption and the corresponding increase in *E*_e_. That is to say, *E*_M_ increases, and *C*_M_ decreases with the increase in strain rate, for the EVA foam with a given density.

### 4.3. Energy Absorption Diagram

Energy absorption diagrams are used to evaluate the optimal energy absorption capacity of cushioning packaging materials with different densities at different strain rates under a certain stress level. In cushioning packaging design, through energy absorption diagrams, the geometric dimensions and the most suitable density of cushioning materials can be optimized and chosen [20,23]. So, it is very valuable to draw the energy absorption diagrams of closed-cell EVA foams for cushioning packaging optimization design. In this section, we depict concrete energy absorption diagrams of EVA foams.

#### 4.3.1. Influence of Density

**Figure 12:**
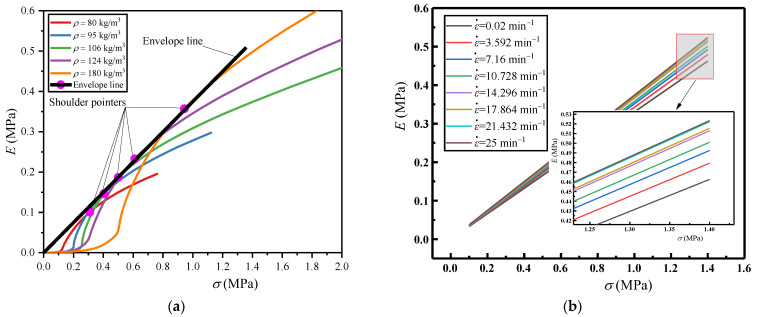
Energy absorption diagrams of EVA foams: (**a**) *E*−*σ* curves and their envelope line of EVA foam with different densities under a certain compressive strain ($\dot{\varepsilon }$ = 25 min^−1^); (**b**) Envelope lines of EVA foams under different compressive strain rates.

Under a constant compressive strain rate ($\dot{\varepsilon }$ = 25 min^−1^), the measured *σ*−*ε* curves of EVA foams with different densities are processed to obtain the corresponding *E*_e_−*ε* and *E*−*σ* curves according to the above methods in Section 3. From the *E*_e_−*ε* curves, the *ε*_O_ values are firstly obtained; corresponding to each *ε*_O_, the *σ*_O_ and *E*_O_ values are obtained from the corresponding *E*−*σ* curves, to determine the shoulder points of the *E*−*σ* curves, as shown in Figure 12a. These shoulder points correspond to the optimal energy absorption capability of EVA foams with different densities under different allowable stress levels of *σ*_O_ and a constant compressive strain rate ($\dot{\varepsilon }$ = 25 min^−1^). Connecting the shoulder points to form the envelope line of all *E*−*σ* curves, this is approximately a straight line, through which the density of EVA foam materials can be matched. Since the *σ*_O_ and *E*_O_ values also tend to zero when the EVA density approaches zero, so this envelope line passes through the origin (Figure 12a). Therefore, under a certain strain rate, the envelope line of these *E*−*σ* curves of EVA foams with different densities satisfies:

(15) $$E=k_{1}\sigma$$

where *k*_1_ is the relationship coefficient between *E*_O_ and *σ*_O_ under a certain compressive strain rate, which is dimensionless and determined by the cell-wall base material and compressive strain rate of closed-cell EVA foam.

More compression tests were carried out for the EVA foams with the above five densities under different compressive strain rates. Repeating the above processing course of tested results, the envelope lines under different compressive strain rates were obtained, as shown in Figure 12b. Likewise, each envelope line corresponding to a density of EVA foam is approximately a straight line passing through the origin. Furthermore, as the strain rate increases, the slope *S*_e_ of the envelope line also increases, indicating that the EVA material absorbs more energy and has a better energy absorption performance under a higher strain rate and a certain allowable stress level.

**Figure 13:**
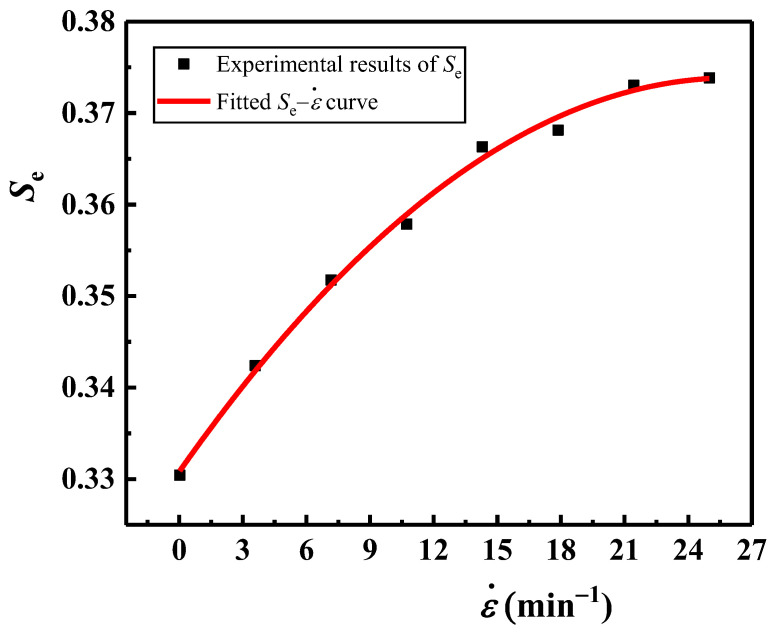
The correlation curve of *S*_e_ versus $\dot{\varepsilon }$.

Using the least squares method, under the strain rates of 0.02, 3.592, 7.16, 10.728, 14.296, 17.864, 21.432, and 25 min^−1^, the fitted empirical envelope line formulas of *E*−*σ* curves for the EVA foams are *E* = 0.3293*σ*, *E* = 0.3464*σ*, *E* = 0.3548*σ*, *E* = 0.36*σ*, *E* = 0.3643*σ*, *E* = 0.3682*σ*, *E* = 0.3732*σ*, and *E* = 0.3751*σ*, respectively. The *S*_e_ values under different strain rates are plotted in Figure 13, indicating that the increase in *S*_e_ becomes slow and tends to stabilize when the strain rate increases to a certain value (such as $\dot{\varepsilon }$ = 25 min^−1^). The relationship between *S*_e_ and $\dot{\varepsilon }$ can be well fitted by a quadratic polynomial curve when $\dot{\varepsilon }$ ≤ 25 min^−1^, and the empirical relationship between them is:

(16) $$S_{e}=-6.5543\;{\dot{\varepsilon }}^{2}+0.0033\;\dot{\varepsilon }+0.3322\;\left(\dot{\varepsilon }\leq {25\;\min}^{-1}\right)$$

When $\dot{\varepsilon }$ > 25 min^−1^, *S*_e_ can be approximated a constant value of 0.3751 as:

(17) $$S_{e}=0.3751\;\left(\dot{\varepsilon }>{25\;\min}^{-1}\right)$$

#### 4.3.2. Influence of Strain Rate

**Figure 14:**
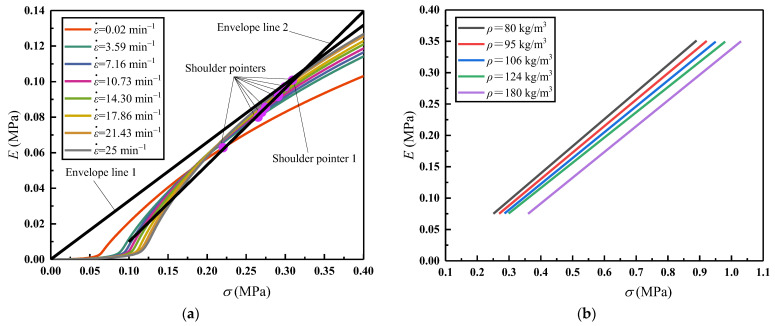
Energy absorption diagrams of EVA foams: (**a**) *E*−*σ* curves and their envelope line of the EVA foam with a given density (*ρ* = 80 kg/m^3^) under different strain rates; (**b**) Envelope lines of EVA foams with different densities.

For the EVA foam specimens with a given density (*ρ* = 80 kg/m^3^), according to the above methods, the measured *σ*−*ε* curves under different compressive strain rates are processed to obtain the corresponding *E*−*σ* curves, plotted in Figure 14a. Similarly, the shoulder points of each *E*−*σ* curve represents the optical energy absorption capability of EVA foam specimens with a given density under different allowable stress levels of *σ*_O_ and different strain rates. Connecting the shoulder points, the envelope line 2 of these *E*−*σ* curves is formed, which is also approximately a straight line, as shown in Figure 14a.

This time, the envelope line of *E*−*σ* curves of EVA foams with different densities under a certain strain rate ($\dot{\varepsilon }$ = 25 min^−1^) is moved from Figure 12a to Figure 14a, viz. the envelope line 1 passing through the origin there. However, for the EVA foam specimens with a certain density (e.g., *ρ* = 80 kg/m^3^ corresponding to the shoulder point 1 in Figure 14a), the *σ*_O_ and *E*_O_ values all decrease when $\dot{\varepsilon }$ < 25 min^−1^. This results in the envelope line 2 passing through these shoulder points (including shoulder point 1) having a larger slope and negative intercept compared with envelope line 1. Therefore, the envelope line of the *E*−*σ* curves of EVA foam specimens with a certain density satisfies the relationship as:

(18) $$E=k_{2}\sigma +E_{b}$$

where *k*_2_ is the relationship coefficient between the *E*_O_ and *σ*_O_ of EVA foam specimens with a certain density under various strain rates, which is dimensionless and determined by the cell-wall base material and density of EVA foam; *E*_b_ is the expected value of the static optimal energy absorption of EVA foam with a certain density, with the unit of MPa and a negative value.

For the EVA foams with densities of *ρ* = 80, 95, 106, 124, and 180 kg/m^3^, based on the tested results of *E*_O_ and *σ*_O_, the fitted empirical envelope line formulas of *E*−*σ* curves are *E* = 0.432*σ* − 0.0333 MPa, *E* = 0.4217 *σ* − 0.0383 MPa, *E* = 0.4122*σ* − 0.0423 MPa, *E* = 0.4022*σ* − 0.0448 MPa, and *E* = 0.4103*σ* − 0.0727 MPa, respectively, using the least squares method based on Equation (18). It can be seen that these envelope lines have approximately consistent slopes, and their *E*_b_ values decrease with increasing densities, as shown in Figure 14b.

## 5. Conclusions

According to the relevant test standards, static and dynamic compressions were carried out on the closed-cell EVA foams with different densities under various strain rates. The obtained results of stress−strain curves, energy absorption efficiency, and energy absorption diagrams were analyzed. The main results and related conclusions are as follows:

(1) The influences of density and strain rate on the *σ*−*ε* curve, elastic modulus, and yield stress of EVA foam are disclosed. Under a certain compressive strain rate, the EVA foam with a higher density has a larger stress and energy absorption, elastic modulus, and yield strength, and the whole *σ*−*ε* curve can be fitted with the Rusch formula. The strain rate does not change the shape of *σ*−*ε* curve and the elastic modulus is not sensitive to strain rate. For the EVA foam with a constant density, the higher the strain rate, the higher the yield strength and energy absorption.
(2) The dependence relationship of maximum energy absorption efficiency and optical strain on density and strain rate were discovered. Under a certain strain rate, with the density increase in EVA foam, the *E*_M_ value first increases and then decreases. Meanwhile, the *C*_M_ value first decreases and then increases, the *ε*_O_ and *ε*_D_ values decrease, but *ε*_O_ becomes closer to *ε*_D_. There is an optimal density corresponding to the maximum value of *E*_M_ and the minimum value of *C*_M_. With the increase in strain rate, for the EVA foam with a given density, the stress in the plateau stage increases. Meanwhile, the *ε*_O_ value also increases, which leads to the increase in *E*_M_ and the decrease in *C*_M_.
(3) Concrete energy absorption diagrams of EVA foams with different densities under various strain rates are depicted. Under a certain strain rate, the optical energy absorption per unit volume on the envelope line of *E*−*σ* curves of EVA foams with different densities is proportional to the optical stress. The change in strain rate leads to the larger slope and negative intercept of the envelope line of the *E*−*σ* curves for the EVA foam specimens with a constant density. But, the optical energy absorption per unit volume on the envelope line still linearly depends on the optical stress.

Based on the tested results, the empirical formulas of elastic modulus, yield strength, optimal strain, and envelope lines and their slopes of EVA foam specimens are derived in terms of density and strain rate. These conclusions and empirical formulas can be used to seek the optimal density and thickness of EVA foam pads in cushioning packaging design. The strain rate range in this investigation is limited; however, the EVA foam materials are often subject to loading with high impact velocities, strain rates, and temperatures. The influences of these loadings on the mechanical properties of EVA foam materials will be further explored in our subsequent research.

## Figures and Tables

**Table 1:** *E*_Y_ and *σ*_y_ values of EVA foam specimens with various densities under quasi-static compression.

*ρ* (kg/m^3^)	*E*_Y_ (MPa)	*σ*_y_ (MPa)
80	2.7734	0.0905
95	4.2659	0.1493
106	5.3702	0.1894
124	5.4784	0.2239
180	6.9843	0.2914

**Table 2:** E* and σ*_y_ values of EVA foam specimens with a given density (*ρ* = 80 kg/m^3^) under various compressive strain rates.

$\dot{\varepsilon }$ (min^−1^)	E* (MPa)	σ*_y_ (MPa)
3.592	3.0046	0.107
7.16	3.0929	0.1113
10.728	3.3872	0.1123
14.296	3.1872	0.1172
17.864	3.512	0.1217
21.432	3.0608	0.1259
25	3.092	0.1291

**Table 3:** *E*_M_, *ε*_O_*, C*_M_*,* and *ε*_D_ values of EVA foam specimens under quasi-static compression.

*ρ* (kg/m^3^)	*E*_M_	*ε*_O_	*C*_M_	*ε*_D_
80	0.298	0.5847	3.3559	0.8821
95	0.3319	0.5789	3.0131	0.86
106	0.3374	0.5746	2.9637	0.8438
124	0.3306	0.5622	3.0244	0.8173
180	0.3181	0.5419	3.1438	0.7347

**Table 4:** *E*_M_ and *ε*_O_ values of EVA foam specimens with a given density (*ρ* = 80 kg/m^3^) under various compressive strain rates.

$\dot{\varepsilon }$ (min^−1^)	*E*_M_	*ε*_O_
3.592	0.3067	0.5758
7.16	0.3111	0.5808
10.728	0.3124	0.5838
14.296	0.3134	0.5841
17.864	0.3149	0.5851
21.432	0.3192	0.5829
25	0.3219	0.5895

## Data Availability

Data are contained within the article.
